# Reporting to audiences in crisis: Disruption, criticism and absent hope in TV journalisms’ rendering of the impactful UK energy price rises

**DOI:** 10.1177/01634437241282921

**Published:** 2024-10-06

**Authors:** Julian Matthews

**Affiliations:** University of Leicester, UK

**Keywords:** audiences in crisis, close suffering, crisis journalism, crisis subjectivity, disruption, energy crisis

## Abstract

How does journalism communicate to audiences who are experiencing crisis? Existing literature suggests that journalists use reporting templates and related practices to report crises with elite narratives and myths (and some ‘disruptive factors’, on occasion). Their news audiences, it follows, are understood as observers of abstracted crisis rather than as those who are experiencing crisis impacts as immediate and affecting. Such thinking becomes challenged by the emerging UK energy crisis. Analysing the corresponding TV journalism shows its reporting responds to several unique disruptive aspects of the crisis (i.e. its ‘seriality’, ‘unpredictability’ and ‘impacts’) rather than reproduce expected myths and authority skew. Additionally, it forms potentially transformative coverage with an included critical commentary on elite inaction and profiteering. Still, omitted at the same time is any relevant explanation and assistance for those audiences affected by the soaring energy costs. The paper argues, subsequently, that this journalism is producing a crisis subjectivity that serves only to reflect back accounts of ‘close suffering’ and uncertainty to audiences, without any sense of hope, support or meaningful action.

## Introduction

This paper examines the reporting of UK TV journalism during a unique energy crisis. The crisis in the UK energy market emerged in 2021, following increases in energy demand (post pandemic), a growing conflict between Russia and Ukraine and impacts from implemented EU and national energy policies. Rapid increases in UK energy prices followed. These produced a 54% rise in the cost of energy for UK residents (over 22 million), positioning 7.5 million of these residents into designated fuel poverty (National Energy Action, 2023) and developed record highs of reported related mental health issues and cost-based company insolvencies (Energy Saving Trust, 2023). Such characteristics meet the UN threshold of a crisis as being a ‘critical threat to health, safety, security, or well-being . . .’ (United Nations International Strategy for Disaster Reduction, 2009) and one that disrupts the social order (Raboy and Dagenais, 1992: 3) rather than an event mediated as disruptive (see Patrona and Thornborrow, 2017). Further, for studies of journalism, the energy crisis produces a time when UK citizens become impacted by unprecedented energy cost rises rather than feature as unaffected and dispassionate viewers. Indeed, those most affected waited 3 months into the reported crisis before hearing the UK government’s proposed actions.

Such a unique situation encourages reflection on the performance of journalism at this time. This paper’s timely response will examine the potentially disruptive nature of the crisis for TV journalism, and its conventions of reporting templates, routinised practices and related characteristics (i.e. myths and authority skew). In then mapping the potential disruptions to the related reporting, the paper will explore what this potentially disruptive reporting is offering for audiences and, specifically, for those who are experiencing real crisis impacts. This combined analysis will allow for greater reflection on crisis types and their impacts on crisis journalism, alongside its inscribed reception.

## The crises that audiences observe

The UK energy crisis provides another example of when energy issues (i.e. involving the production, distribution or cost of energy) visibly break onto the everyday news agenda (Endres et al., 2016). But the observed impacts of the energy crisis on UK citizens in this case make it a unique and valuable reference point with which to observe the performance of its reporting. Previous studies explain that audiences will receive different journalistic accounts of crisis on account of their proximity to the reported incidents. Crises, it follows, are generally judged to be newsworthy events by journalists. But national crises that unfold close to TV audiences – as in the energy crisis for the UK news audience – are considered to be significantly more newsworthy (Hughes et al., 2006). In essence, the journalistic value of these proximate events is expressed in the forms of the continuous reporting that they receive (Berkowitz, 1992), which first chart the disruption to the social order before addressing efforts to restore ‘normalcy’ (Joye, 2014). This order emerges from the reporting templates that journalists apply to continuous coverage at these times (Matthews, 2016). Templates inform the appropriate selection and framing of built-in reported elements over time, including those of event reconstruction and problem definition, representations of victims and acts of assistance, alongside characterised responses from elites (including recognised expertise) as are most acutely observed in crises that follow largescale terrorism incidents (see Matthews, 2016). Watching audiences are thus drawn into this particularised and unfolding mediated account of crisis.

In reality, the audiences’ experiences of mediated crisis form temporally. Reporting introduces the crisis through a reconstruction of the unfolding local or global crisis event (Cottle, 2009). Included here are the thoughts and speculations of relevant included expertise on what is known about the origins of the crisis event (An early account by Walters and Hornig, 1993 charts these as accompanying risk and crisis accounts in the US, for example). Appearing alongside the included voices of journalists and experts are those impacted by the crisis at this point (i.e. victims). Victims voice experiential reactions of crisis impacts and events as Cottle (1993) finds in localised discussions of environmental crises, and in their place ‘add colour’ to reporting descriptions as well as personalise crisis moments for the national audience (Matthews, 2016). All of which, nonetheless, falls short of allowing ordinary people to offer reflexive thoughts – or forms of ‘social rationality’ – on crisis events that Beck (1992) suggests would demonstrate a more progressive discussion of crisis. Subsequently, the thoughts of those audiences watching the unfolding crisis become shaped by the commentaries of journalists and the perspectives of the elites they include.

Following efforts to reconstruct the crisis event, traditional reporting extends its commentary on the developing crisis. Often present are reproduced mythic themes which naturalise forms of crisis as inevitable and applicable to certain geographies or times, or as emerging as a consequence of simplistic causes which Miller et al (2015) find in coverage of ecological crises. Likewise, as Myers (2021) observes in the reported UK financial crisis, reporting regularly reduces the crisis to the parts that ‘heroes’ and ‘villains’ play. It also situates large scale crisis events in terms of a voiced ‘national resolve’ (Joye, 2018). National crisis coverage often includes elements of sympathy, solidarity and calls for action (see Schudson’s, 2002 discussion of the 9/11 crisis reporting, for example) and includes political elites as an informing discursive presence. As Cooper et al. (2021) observe in the recent European migrant crisis reporting, media narratives regularly follow features of political elite commentary. These reproduce, often uncritically, elite spoken efforts to frame the crisis, the crisis response and what follows. Some developing crises – such as the recent COVID 19 pandemic – position journalists as reliant on government information and analysis (Perreault and Perreault, 2021). Others, however, show journalists as actively reproducing political elite crisis commentary and allowing for elites to successfully manage, or even political appropriate, them (both Bennett et al., 2007; Klein, 2007, discuss this prominent feature across various crisis events, for example). Audiences’ understandings of crisis, it follows, become contained within these managed representations.

But not all crisis reporting forms in this way or, in turn, directs its audiences to particular managed reactions. Crises can be unpredictable in their timing and contents, as is the case of the emergence and impact of the UK energy crisis. Such unpredictability can wrongfoot political elites and, in these moments, allow opportunities for those external to the political establishment to ‘capture the attention of media and public’ as Blondheim and Liebes (2002: 275) observes in crisis coverage of political violence. Whereas alternative commentaries can emerge when elites’ crisis responses become delayed, as Durham (2008) notices in the aftermath crisis coverage of Hurricane Katrina in the US, other disruptive factors can impact coverage and intervene in the journalistic representation of crises. Disruptions – based on geography, expertise and commentary as experienced in terms of a significant recent tragedy in the UK (Matthews, 2023) – can alter the fabric of coverage to include what Cottle (2006) describes as resonate symbols, dramatic visualisations and embedded emotions. Providing space in coverage for challenger groups voices, criticism of elites and expressions of anger (Pantti and Wahl-Jorgensen, 2011 suggest the latter forms – to various degrees – in reporting of UK tragedies), this reporting is able to encourage contrasting ‘public solidarities on the basis of collective sentiment’ (Cottle, 2006: 415).

Further, the ongoing debate over the character of crisis reporting is reflected in those integrated assumptions about its relationship with news audiences. Studies view the inscribed news audience through the lens of the political subject. Reporting of crisis, it follows, either invites them or not, ‘to act and fulfil their role as citizens’ (Nikunen, 2019: 10 – Nikunen reflects on the reporting of a tsunami in this case). Most often the crisis reporting replete with elite narratives and reduced political positions serves to delimit audiences’ understanding of crisis, as Temple et al (2016) detect at times of economic crisis. It can even act to inculcate audience beliefs such as those of ‘decisions that affect them personally are out of their control. . .’ which Katz Jameson and Entman (2004: 38) observe during a US budget crisis. Or, moreover, such reporting can serve to legitimise existing processes and practices and the wider political and economic systems that support them as is characterised in the reporting of the financial crash in 2008 (see Berry, 2019). But at the same time that reporting moulded or (politically) managed may be shaping public beliefs in the absence of contested knowledge (Happer and Philo, 2013 recognise this in the reception of the climate crisis reporting, for example), other reporting that offers disrupted coverage may be doing the same. Cottle (2006) suggests that disruptive coverage can be ‘transformative’ for audiences. Rather than trivialise unfolding crises or create media spectacles of them, disrupted coverage has the potential to ‘stir consciousness and deepen public understanding’ (182). Studies that illustrate a ‘moral-ethical turn’ in media enquiry develop this point in relation to audience engagement with crisis impacts. Significant is how reporting offers crisis victims forms of visibility (both Chouliaraki, 2006; Ong, 2009 discuss its presence following disasters) and space for their performance of context-based suffering which Chouliaraki and Stolic (2017) observe in selected visuals that accompany reporting of the 2016 migrant crisis and those which arguably form during the recent pandemic. Such features can encourage audiences to adopt different understandings (Pantti, 2019), moral positions (Höijer, 2004) or even potential solidarities / political responses (Joye, 2014). Still, these combined studies continue to see the watching audiences as attentive observers of crisis events rather than consider them as crisis participants.

The above review discusses the characteristics of crisis reporting that audiences will observe. Emerging crisis reporting, we note, can reflect applied reporting templates alongside their mythic representations, access to limited voices and authority skew (as is evidenced in various crises from those focused on ecology, disasters, terrorism, migration, finance to the pandemic). In the same way, sometimes the location, widespread impact and related evidence of local suffering of some tragedies and extended events can produce aspects of disrupted and transformative coverage. Nonetheless, studies agree that whatever elements of crisis are being reported, such elements are shaping the understanding of the watching news audience who – in this thinking – remain as distant and emotionally removed from the impactful crisis. On reflection, this academic understanding of the crisis audience overlooks the importance of the experience of the crisis context and any crisis participation to audiences’ understanding of crisis coverage. As the latter appear important within widespread and impactful local crisis events – such as the UK energy crisis, we need to incorporate these features into our academic assessments of the performance of reporting. Reflecting this new thinking about crisis journalism and its audience, the following question informs this paper’s analysis of the TV reporting: How is the emerging UK energy crisis configured in TV journalism and what does this offer for an audience ‘in crisis’?

## Researching energy crisis reporting

In response to the above question, the paper explored the reporting of the energy crisis across UK news broadcasting. TV news broadcasting was included on account of its overall reach and for the levels of trust to which audiences ascribe TV news generally (see Newman et al., 2020). News programmes were sampled to incorporate coverage spanning the first substantial increase in energy costs and related mediated discussion (i.e. from 1 April to 30 June 2022). The project used the *Box of Broadcasts* database to collect the following evening news programmes (selected for their extended length and perceived seniority in the news diet) across the period: BBC News (*n* = 44), ITN News (*n* = 26), Channel 4 News (*n* = 29), Channel 5 News (*n* = 36) and Sky News (*n* = 39). The included sample was cleaned for duplicates before being analysed using content analysis. Insights from both the crisis literature and an inductive overview of the collected coverage informed a general analysis of (i) story themes, (ii) included speakers and (iii) the presence of explanations and solutions. Several general story categories were devised and used as a consequence, including: (i) crisis development, (ii) crisis impacts, (iii) crisis support/ advice, (iv) crisis solutions and (v) crisis politics. At the same time, sampled voices – either those quoted or referenced by journalists – were included to complement the analysis of themes. These were coded in their original form before then being recoded into several groups, including those of: politics, energy professionals, charities, businesses, ordinary people, other professionals and regulators. A second stage of the analysis followed, guided by the desire to produce a nuanced grasp of the mediated energy crisis. This examined the relationship between the recorded content and voices and, in the process, recognised the organised nature of the interactions between them. The intention was to understand the various ways that this uncertain and unpredictable energy crisis was being professionally understood and then communicated to TV news audiences. The process involved surveying both story content and included voices within each theme which allowed the study to identify the communication of several dominant understandings of the energy crisis, including: intensity ( in crisis developments stories), struggle and suffering (in crisis impacts stories), calls for action (in crisis solution stories), criticism (in crisis politics stories) and support as discrete aspects of the developing energy crisis. As will be explained below, these reflected journalists, and the included voices, efforts to make sense of a largely uncertain and impactful crisis.

## A media tale of uncertainty and disruption

**Figure 1.**
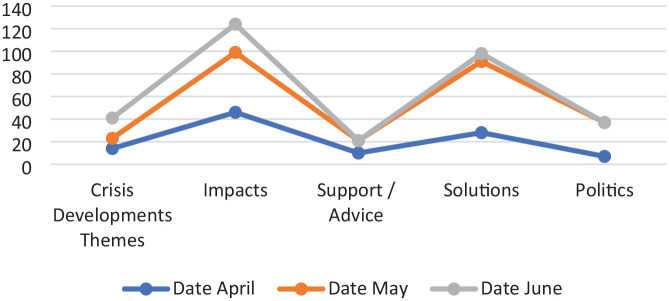
Themes by date (month).

The analysis reveals how the emerging TV journalism makes the unusual developments of the UK energy crisis comprehensible for its audience. Taken at face value, this coverage outlines a common structured response to the energy crisis. While this structure offers similarities to previous observed journalistic responses, it appears notably different in the frequency of its reproduced elements of crisis developments and impacts and related politics, solutions and advice (see Figure 1). Although it is plausible that such a reporting structure can incorporate potential mythic elements and authority skew, a closer exploration of it reveals much more.

A detailed analysis demonstrates that the energy crisis reporting breaks with previous prevailing academic expectations. A significant difference is the series of extended crisis developments that feature across each of the selected months (April, May and June). This pattern contrasts with the normal reconstructed singular crisis event, and the staged efforts to resolve related crisis impacts that follow (see Matthews, 2016). Story themes, appearing across the 3 months, focus on crisis impacts and crisis solutions (in greater frequencies in May and June), packaged between lesser numbers of crisis developments, support and politics themes. This formation departs from the recognised elite focused coverage in traditional crisis coverage (offering forms of elite managing or expert certainty – see Bennett et al., 2007) and the presence of a simple mythologising of crisis, its impacts and those involved (see Temple et al., 2016). A journalistic reaction to an unpredictable and evolving crisis appears instead. This continually monitors developments (i.e. unpredictable price rises), recounts related impacts and, in between which, it adds limited reflective commentary on what can be done (support, solutions and wider political discussion).

**Figure 2.**
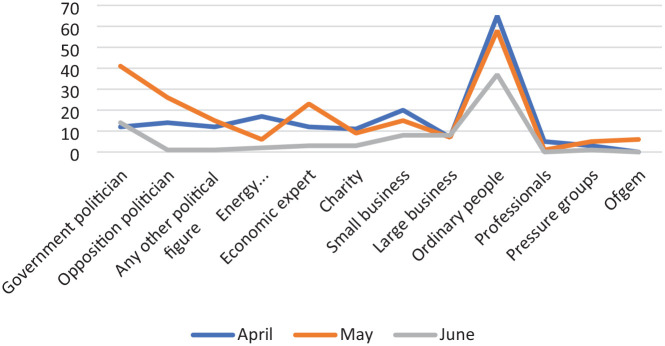
News voices by date (month).

This unique journalistic response is reflected also in the news access opportunities that the coverage provides (see Figure 2). Among the recorded voices, ordinary people (*n* = 164), government politicians (*n* = 60), business owners (*n* = 57), opposition politicians (*n* = 43) experts (*n* = 38) feature in that order of frequency. This configuration challenges existing notions of the dominance of elites in coverage and, therein, their ability to define and to potentially appropriate the crisis in the role of ‘crisis definers’ (Bennett et al., 2007). Included in the stories on crisis impacts and crisis developments are ordinary peoples’ voices and those of business representatives who appear routinely to offer thoughts on, and reactions to, the energy price rises. Experts appear, alongside energy company representatives and some politicians, to explain the complexities of potential solutions. Politicians feature also in the theme of the politics of the energy crisis but as mainly engaged in arguments between the reproduced political parties. Observing the detailed interconnections between the voices and the themes in the reporting (see Table 1) helps to further develop a picture of what is being consumed by the watching audiences (who are also experiencing the described energy cost rises). In the absence of political elite management, mythologized coverage or elite reassurance, we begin to see repeated messages of (i) intensity and (ii) struggle and suffering, alongside some (iii)criticism and (iv) support over this time.

**Table 1.** News voices by story theme.

		Government politician	Opposition politician	Any other political figure	Energy company representative	Economic expert	Charity	Small business	Large business	Ordinary people	Professionals	Pressure groups	Ofgem	Total (%)
Theme	Crisis developments	5	3	3	9	9	5	4	6	37	3	3	3	90 (19)
	Impacts	16	7	17	5	14	13	28	12	93	3	2	0	210 (46)
	Support	0	0	0	4	2	4	0	1	3	0	0	0	14 (3)
	Solutions	35	24	8	6	12	1	3	3	20	0	4	3	119 (26)
	Politics	4	9	1	1	1	0	0	0	11	0	0	0	27 (6)
Total		60 (13%)	43 (9%)	29 (6%)	25 (6%)	38 (8%)	23 (5%)	35 (8%)	22 (5%)	164 (36%)	6 (1%)	9 (2%)	6 (1%)	460 (100)

## Intensity

Crisis development stories punctuate the observed coverage. Collectively, they communicate the intensity of the energy crisis via (i) the regularity of reported crisis developments, (ii) a represented uncertainty among those with expertise to know and (iii) the level of represented crisis impact. As explained, the reporting of the UK energy crisis emerges in a series of disruptive crisis events (i.e. price rise announcements / discussions thereof ) which sidestep the standard journalistic crisis response. Forming differently to those stories based on singular crisis events, these stories focus on the ongoing and inescapable crisis. In essence, reporting is marked by new, and anticipated, price rise announcements and commentary on their impacts (see Figure 1, – April 1, 19, 27, May 24, 31 and June 9, 10, 15, 25). On occasion, such stories feature additional related themes on crisis impact, solution and support. But these additional themes never engage with crisis resolution or introduce actions and responses common to the forms of crisis resolution that are found in mediated singular crisis events. Rather, ideas of an uncertain energy crisis prevail, and these ideas negate the other expected understandings and responses commonly found in event-based crisis reporting.

Furthermore, the story descriptions of crisis are replete with a sense of intensity. From the first stories, the reporting references the crisis as unpredictable and unusual. BBC News, for example, begins its coverage with the phrase ‘The biggest rise in energy prices in living memory has come into effect in Britain, just as millions of households are dealing with a surge in the overall cost of living’ (BBC News, 1 April). Evidence of crisis impacts, alongside expressed uncertainty over the causes and developments of the crisis structure this news commentary. Notably absent are the reliable experts and their speculative certainty over crisis events that commonly inhabit crisis event reconstructions (see Walters and Hornig, 1993). Against this context, Broadcasters ask ‘How did we get here? Why is all this happening?’. In this BBC example, the BBC Business Editor responds to the news anchor’s questions with: ‘I think the cost-of-living crunch we’ve been talking about for weeks started to hit home today. But the origins of it are many months ago’ (Simon Jack, BBC Business Editor). Providing a rare explanation that incorporates several informing causes in what follows, the editor’s account takes a place alongside a limited number of other explanations found across the coverage (i.e. war in Ukraine *n* = 27, global energy markets *n* = 10 and the pandemic *n* = 7). The general absence of experts and their explanations in the coverage illustrates a disrupted reporting that cannot grasp and predict the direction of the ongoing energy crisis. What is communicated to audiences, instead, is an overwhelming sense of uncertainty.

The theme of intensity is reflected additionally in discussions of crisis impacts that appear in these crisis development stories. Communicating the depth and breadth of crisis impacts across Britain becomes central to understanding the developing crisis. These provide a sense of the scale of the crisis impacts and address the watching audience as a community affected – both of which provides a contrast with previous crisis coverage. In introducing forms of ‘close suffering’ of UK citizens ( in contrast to distant impacts or suffering of faraway others observed elsewhere – see Chouliaraki, 2006), reporting allows victims to voice their experiences. Victims’ reactions, it follows, appear most frequently (ordinary people, *n* = 37) within the journalistic commentary of crisis developments and its included professionals (i.e. energy company, *n* = 9 and economic expert, *n* = 9).

## Struggle and (close) suffering

In context of the observed reporting of the intensity of crisis developments and combined uncertainty from absent expertise in the evolving coverage, TV journalists include dedicated accounts of crisis impacts. Impact themed stories appear frequently in the period (*n* = 124) and, at times, connect to related themes on solutions and support/ advice. Subsequently, crisis impacts become a significant, rather than a predictable and limited, aspect of this crisis coverage and a feature that focuses reporting away from any potential mythologising of crisis impacts or victims’ situations. Within the created space, included stories detail the struggles faced in crisis moments and, in turn, provide opportunities for relevant and accompanying voices and reactions. These include those associated with the ‘close suffering’ of the most vulnerable at this time. While the voices of ordinary people and business owners predominate in coverage (*n* = 93 and 40, respectively), stories include some lesser speaking opportunities for political figures (*n* = 17), economic experts (*n* = 14) and charities (*n* = 13) in supporting discussions of observed impacts.

Moreover, the idea of struggle is introduced as an outcome of the unknowable crisis in this coverage. Broadcasters communicate the combined intensity of crisis and its implication as part of their introductions to crisis impacts. Channel 5 News, for example, explains the energy crisis as ‘. . .A daily struggle with no solution in sight’ (Channel 5 News, April 6). These introductions build accounts of crisis impacts and therein locate the importance of observed struggle in their descriptions of time (i.e. daily), likely duration (i.e. endless) and in terms of an impact geography. In addition to offering accounts of the severity of the energy crisis, journalists explain crisis impacts via a widespread geography that encompasses the household, the local and the national community. In doing so, the coverage represents these levels with case studies of the experiential struggles of individual homeowners alongside the owners of local businesses and national industries.

Ordinary people are used to detail the specific impacts of the developing crisis in this coverage. Their featured experiences recount crisis impacts on ‘ordinary’ households and often their accompanying expressions of helplessness feature as headlines and introductions to stories. A BBC story appearing midway through the sampled coverage explains, for example – ‘Beth from Bristol and her family are like millions of others, seeing their bills spinning out of control . . .’ (BBC News, 24 May). The recounted experiences of Beth – and others – in the coverage document the newfound experience among ordinary people of a lack of financial control in stark contrast to their controlled and managed finances in pre-crisis times. Scenes from across the country of ordinary families’ worries over escalating finances (often articulated as working couples with children) document the widespread impact geography of the crisis. By focusing on the vulnerable from low-income families, the elderly, disabled and the unemployed also, this coverage outlines the depth of geographical impact in addition to that of its breadth. David from Sheffield is introduced at the start of the crisis coverage as being forced to avoid using any gas and using electric sparingly, for instance. In reflecting on his experience, he says:

We are living in the 21st century, and I feel as if I am going backwards and doing things that, you know, probably what you would hear people do in the olden days. (BBC News, 3 April).

Later, the included ordinary voices speak of having to make decisions over either buying food /eating or heating their homes that day. Appearing alongside these vignettes, charity representatives document the reach of the crisis and the observed commonality of these desperate experiences, including the impact on ‘ordinary’ working people as is explained here:

People are proud. They work full-time. They still can’t afford it. There was a lady in here close to tears. She just didn’t know what to do. People have not found themselves in this situation before. (Charity representative, ITN News, 13 April).

The included historical comparison and expressions of desperation here illustrate the reporting of suffering in modern Britain at this time. Such episodes encourage us to reflect on these examples of ‘close suffering’ and what reactions these would produce among the watching (local) audiences.

Additionally, TV journalism widens an account of the crisis geography to include local businesses and national industries. The experience of local businesses represents impacts on the fabric of the local community. Owners of cafes, hairdressers and other small enterprises speak of their concerns about and experiences of, the crisis in contrast to pre-crisis, ‘better times’. The overwhelming emotion that features is one of anxiety over their ability to cope and continue trading – as is observed in this fear-based response from a café owner responding to the question ‘why are you scared?’: ‘Because I don’t want people not coming. I’ve worked really hard for nearly 2 years to build this place, and I’m afraid, by putting the price up too much, people will go elsewhere’ (Café Owner, Sky News 30 May). These included local business narratives reflect commonly on the basic survival of the local business and consequences for the local community (including impacts on their suppliers and customers). Further, it is a configuration that continues in the reporting of large national industries. In one case, a Nursery Operations Manager explains the experience of ‘astronomical’ energy prices impacting on their growing of food for the UK market:

Gas has always been a big expense in a nursery of this size, but in the last six or nine months, it has been astronomical. The price increase we have seen in the last 12 months has gone from forty pence per therm to four pounds and even hit eight pounds per therm on a day price . . . (Sky News, 20 May)

In the story, the manager illustrates how the nurseries’ decision to grow food has significantly undermined the ability of the business to function at a time when other businesses decided to not grow food on account of the aforementioned ‘astronomical’ energy prices. Naturally the outlined impacts are reflected in the gravity of these comments, alongside others provided on a range of other industries struggling in the face of energy costs. One Sky News reporter summarises the fundamental damage to this network of industries in the UK when suggesting that the energy crisis: ‘. . .is economically destructive. People can’t do the work they want to do . . . It is not just a cost of living crisis, it is a way of living crisis. . .’ (Sky News, 20 May). The aforenamed ‘way of living crisis’ for both households, local business and national industries pre-empts reported calls for action across the coverage.

## Calls for action

As we have heard, TV journalism communicates this severe crisis through intense developments, a geography of crisis impacts and accounts of related suffering. What is offered here breaks clearly with established ideas of existing journalistic reactions to the singular crisis event. In addition to characterising features of the developing crisis, reporting also includes mediated solution themes. These, as the second largest number (*n* = 98) include the voices of government (*n* = 35) alongside those of opposition parties (*n* = 24), ordinary people (*n* = 20), energy companies (*n* = 6) and businesses (*n* = 6). Observed at face value, solution stories appear as a logical place to find instances of government representatives directing the crisis narrative and even appropriating the situation for their ‘political ends’ (see Klein, 2007). The expectation of a present political-led narrative further grows when we note that reproduced ‘calls to action’ characterise the included solution stories.

On inspection, however, these calls for action emerge from those other than government and any crisis leadership attempt. The prominent voices on this matter are those of energy companies. These take a lead in calling for imminent solutions while also explaining their view of energy price rises in context of a volatile energy market in this coverage. In the absence of an established political narrative on what is to be done, such calls appear prominently. As the following example in Channel 5 news explains:

Keith Anderson (CEO, Scottish Power) has called for 10 million households to have their bills reduced by £1,000 in October, when another rise is expected. He says unless this happens millions face a horrific winter. (Channel 5 News, 9 May).

Energy companies’ ‘calls for action’ remain central to this developing solution-based crisis reporting. Such instances demonstrate the pro-active public relations strategy of these companies and their greater communicative agility over that of the UK government during the growing politicisation of the energy crisis. Their reported comments express concerns about escalating impacts before then championing a preferred solution of government intervention in contrast to other solutions being offered elsewhere (i.e. a windfall tax on themselves as energy companies)

As indicated, these stories also recognise the absence of a government strategy. Through the energy crisis coverage, the government are represented as purposefully lacking a clear narrative and position on what is to be done despite acknowledging an unpredictable crisis and its impacts. Minister Kwasi Kwarteng’s widely reported comment – offered below – reinforces this ongoing stalled position:

Both the Chancellor and Prime Minister said there is more to do, and we have to just wait and see what is forthcoming . . .It’s a difficult time and people are under huge stress. We also know the cost of living is a very real issue and nobody is suggesting that the government can pay the entirety of the energy bill. What we are committed to is giving support. (BBC News, 24 May)

The commitment of government support emerges for the first time in May, following only a basic recognition of support being required up to that point. As others observe elsewhere (see Durham, 2008), the absence of consistent commentary from political leaders on the crisis produces space for energy companies views and others to appear in this case. In addition to energy companies, opposition parties feature and offer the suggestion of a windfall tax on energy companies as an alternative solution to the crisis. Opposition political parties not only gain discussion of their alternative policy – albeit in contrast to energy companies’ solutions – but they are provided with space to elaborate simultaneously on observed government inaction at this time. The following comments of Labour MP, Lisa Nandy – demonstrates this:

What we’ve got with this government is a group of people who aren’t just out of touch but are living on another planet. This is a crisis facing every family and business across this country. We need to get money back into peoples’ pockets. (Channel 5 News, 5 May)

Criticism of government inaction continues through to June, when the government announces an Energy Bill Support Scheme (i.e. a £400 grant to support households). From that point, reporting is redirected and follows the suggested policy through the parliamentary procedure, starting with the Queen’s speech in the UK parliament.

## Criticism

As noted, the reporting of the energy crisis lacks identifiable government crisis leadership and reproduces content and voices different to what is expected. This dynamic appears also in the included political themed stories. Political themed stories feature significantly less than solution themed stories (*n* = 37 and 98, respectively). Still, these exemplify – in their place – a charged political atmosphere and those criticisms levelled within it. The UK government appears to speak infrequently here (*n* = 4) and only to rehearse comments on all-pervasive global forces as restricting what is possible in the situation. Based on a summarised precarious position of the UK government, these stories rehearse criticisms and alternative policies from opposition parties’ voices (*n* = 9) as is summarised in the following example from Channel 4 News:

The government is being shouted at from left and right, by Conservatives demanding tax cuts, and Labour demanding an emergency budget. The Chancellor is warning tonight of a tough few months, saying he can’t make global forces disappear. (Channel 4 News, 18 May)

This coverage incorporates voiced criticism of both government intransigence and their self-proclaimed powerlessness in the face of the global energy market. It is also directed to the activities of other players in the energy crisis – that is, the energy companies– in the time before the announced Energy Bill Support Scheme.

Discussion of the politics of the UK energy crisis includes consistent criticisms of the actions of energy companies in the crisis. From the start, reporting includes energy pressure groups criticising these companies’ handling of, and responsiveness to, energy customers and their problems. Later, crisis coverage visibly reassesses the place of these companies in the crisis following their business announcements of record profits. This begins with reported reactions to the announced profits of British Petroleum (BP), from January to March 2022, as in this example:

It will be an irony not lost on many people that while the majority of households are trying to get to grips with soaring energy bills, one of the energy giants has just notched up soaring profits. And a major factor underlying quarterly profits for more than a decade, some £5 billion, is rocketing oil and gas prices . . .. Labour, the Lib Dems and the Green Party want a windfall tax on BP and North Sea oil producers, calculating it would raise £2 billion. (ITN News, 3 May)

Similar stories develop the idea of these companies’ profiteering from an extended crisis. Reporting features companies expressed ‘surprise’ at levels of the observed controversial profits followed often by the main opposition parties’ retorts to these public relations responses. One such from the opposition party, Labour, suggests: ‘Oil and gas companies in the North Sea made more profit than expected. So, let’s have a windfall tax to help people with energy bills with up to £600 for those most in need’ (Labour Leader – Kier Starmer, ITN News 3 May). Featuring in greater number in both May and June, these stories sustain a collective critical commentary on the inactions of both government and energy companies. Also, these lay a foundation on which reporting discusses the need for support in the face of escalating crisis impacts.

## Support

As has been explained, the unfolding coverage charts the developing crisis with accounts of crisis intensity, examples of suffering, calls for action alongside criticisms. Included also are a smaller number of commentaries that are devoted to addressing the need for, and evidence of, support during the crisis. Reported discussion of support falls into two categories, including (i) calls for, and evidence of, crisis support and (ii) actual provided crisis advice. As for the first category, crisis coverage traditionally includes comments on the support needed by crisis victims. Formed often from announcements from charities such as ‘Charities say there’s been a surge asking for their help’ (Channel 5 News, 29 April) from Channel 5 News, these stories explain the growing demand for assistance from those affected, as evidence by volunteers on phone help lines and those working on-the-ground. Some include examples of ordinary people, charting their experienced problems with communicating with energy companies and evidence of miscommunication. Others feature charities explanations of aiding those affected, including their hosting of warm spaces and their offering of warm meals etc.

The second aspect of coverage extends the focus to include ‘crisis advice’ for audiences. Located periodically across the coverage, stories feature the voices of energy companies and energy poverty charities who explain the practicalities of reducing energy consumption. The Energy Saving Trust – as an example of the latter – is mentioned in the following summary example:

Well, the Energy Saving Trust say a standby saver or smart plug allows you to turn all your appliances off standby in one go. Money saving tips include turning off the lights when you’re not in the room, have a quick shower, and for those of us who love popping the kettle on, they say don’t overfill the kettle —only boil the amount you need. . . (Channel 5 News, 27 April)

Such comments are mechanically reproduced as part of the narratives of these solution-themed stories. Pinpointed often are potential energy saving activities that serve in effect to reduce only small amounts of energy consumption. Noteworthy is how these commentaries on limited energy saving practices emerge as disconnected from the other surrounding discussions of severe crisis impacts and the accompanying everyday struggles. At best, some forms of reflection in this respect appear as part of joined-up thinking on such advice and severe crisis impacts. The following financial journalist offers a rare example, here:

Frankly there are lots of little things we can all do to reduce our energy consumption, so that will bring down our bills a little. However, it is little bits here and there - it is all helpful, but we really need to get big help, government level help, because most people’s energy bills have more than doubled in the last few months, more than most people can cope with. But it’s still a way to save some money and right now, particularly, every penny counts. (Financial Journalist, Channel 5 News, 27 April)

In sum, these accounts of crisis support simply reflect messages of limited assistance for the impacted alongside what appears to be tokenistic energy saving advice.

## Conclusion

This paper has explored TV journalisms’ response to an unexpected ‘fateful event’ of late modernity (Giddens, 1992). The unexpectedness and severe impacts of the UK energy crisis, as a ‘fateful event’, requires TV journalism to speak to audiences as those being affected by the crisis. What emerges from the analysed TV journalism is a challenge to our existing understandings of the prevalent, standardised and disruptive crisis reporting. Its observed elements provide a new structured crisis coverage which include, intensity, impacts, calls for action, criticisms and support. Moreover, several disruptive factors that are unique to this crisis underpin the coverage and its reporting practices, including those of: (i) seriality, (ii) unpredictability and (iii) widespread impact. The connections between these disruptive factors and the produced coverage significantly extend our grasp of the content and the production of existing crisis reporting.

As such, the paper’s analysis of coverage shows the importance of the ‘seriality’ of the energy crisis. As a series of crisis events, the energy crisis evades journalisms’ traditional staged response to singular events (which often involves mythic elements, specific expert opinions and political elite commentaries – see Matthews, 2016) or its reaction to the sometimes unexpectedness of a singular crisis event (see Blondheim and Liebes, 2002). More specifically, this developing energy crisis avoids the often-witnessed political elite efforts to discursively capture reporting of the single crisis event. What is more, a second disruptive factor – the ‘unpredictability’ of the energy crisis – appears to compound this process. As shown, experts and elites are represented as unable to predict and discursively manage the reported crisis developments (i.e. the escalating energy prices). Emerging, in the space that this creates, are ‘calls for action’ from others and sustained criticisms focused on government intransigence, and to a lesser extent, on energy companies’ profiteering. Further, evidence of widespread and escalating crisis impacts provide a final disruptive factor. Corresponding coverage on the impacts from rises in energy prices detail the ‘struggles’ of ‘ordinary’ people, local businesses and national industries, alongside the ‘close suffering’ of the most vulnerable in the UK. While a discussion of crisis support is encouraged by these disruptive factors, related reporting includes only modest suggestions for energy reductions rather than those proportionate to the recorded level of crisis impact. In sum, the reporting of the energy crisis shows an emerging dynamic between (i) a fluid, unpredictable and impactful crisis and (ii) the communicative space of reporting and what journalists, politicians and experts can provide at this time.

Additionally, this account of the reporting of such a fateful event has added implications for our understanding of the inscribed crisis audience. Explored from the perspective of audiences observing mediated crisis, the examined reporting includes critical and challenging elements. Despite ignoring relevant issues with the wider economic system and its reliance on fossil fuels, this reporting questions elite thinking, explanations and (in)actions and promotes potential solidarities with those represented as experiencing crisis impacts – all of which are potentially transformative characteristics (see Cottle, 2006). Nonetheless, this view obscures the implication of those watching audiences as being ‘in crisis’. Ignored in the above, therefore, is a sense of the ‘crisis subjectivity’ that this reporting of crisis complexity, severe impacts, intransigent elite responses, alongside a lack of solutions and advice provides for impacted audiences. For those for whom the energy crisis is undermining their ability to pay for energy, and in turn, to cook and stay warm (i.e. destabilising a present and near future sense of stability / ontological security – Giddens, 1992) the observed TV journalism appears engaged in a ‘symbolic annihilation of hope’ at this time.

Finally, we can note that the reported developments have the potential to generate new academic interest in the relationship between crisis, journalism and audiences in several ways. First, based on what is found here, discussions about mediated crisis should now look to incorporate and reflect on, the form of the unpredictable and evolving crisis observed here (as opposed to the more common singular event-based crisis) and its implications for journalists’ practices and reporting. Relatedly, second, we need to assess, with relevant journalists in interview, how their differing reporting structures and templates become adopted over time and adapted when facing various crisis types. Lastly, we need to reflect on our existing understandings of the inscribed news audience and, importantly, acknowledge audiences who are actually experiencing crisis. This requires research to revisit the ‘crisis reception moment’ and think about the forms of crisis subjectivity that journalism produces at such times. Of interest would be to explore what emerges from when audiences’ lived experience of crisis impacts, intersects and interacts with those interpretations and understandings being consumed from crisis reporting, including the latter’s representations of elite critique, ‘close suffering’ and delimited solutions. Collectively, such research would allow for greater academic and journalist /professional reflection on both those actual, alongside those ideal, performances of journalism during crisis moments.
